# The Local Treatment of Acute Follicular Tonsillitis

**Published:** 1895-04

**Authors:** A. B. Farnham

**Affiliations:** Milwaukee


### THE LOCAL TREATMENT OF ACUTE FOLLICULAR TONSILLITIS.

BY A. B. FARNHAM, M. D., OF MILWAUKEE.

*Mr. President and Doctors:* Your officers were promised that the papers of this section should be brief and to the point. To secure this the tonsils were selected for the bulk of attention and divided up among us. The Chairman will endeavor in the three minutes allotted to him to set an example. The object of local applications is to cut off totally or to interfere as much as possible with the supply of ptomaines to the general system. If the supply can be cut off entirely the disease is aborted and ends as soon as the poison absorbed has been eliminated. The method my experience makes me most approve, is to apply locally to each evidently affected follicle a solution of super-saturated alcoholic solution of iodine. The method of application is with a very minute pledget of cotton on the end of a probe, certainly no larger than a No. 6 wire. Apply only a short distance within the follicle. Follow with a deeper application of No. 1 solution of iodine and glycerine. Apply to all follicles presenting white spots at orifice. To those not presenting, it is well to apply the No. 1 iodine and glycerine solution. Reinforce the tonsillar applications by rubbing the tongue with same solution, No. 1; and in some cases also make a post nasal application of same. If only one tonsil is affected, always rub the surface of other and introduce a little iodine into the larger crypts. If any crypts evidently contain hardened secretions, I make a brief effort to cleanse them with a syringe specially adapted to the purpose.

### DISCUSSION.

*Dr. Neilson:* I have never used the the treatment advocated, but I have treated tonsillitis on much the same principle by a solution of nitrate of silver, and obtained about the same results; the idea being to destroy the infectious germs present.

